# Rhabdomyolysis Induced Acute Kidney Injury in Hyperglycemic Hyperosmolar State Patient With New-Onset Diabetes: A Case Report

**DOI:** 10.7759/cureus.9188

**Published:** 2020-07-14

**Authors:** Qasim Khurshid, Laila Khalid, Norina Usman, Neelam Neupane, Anas Mahmoud

**Affiliations:** 1 Internal Medicine, Xinjiang Medical University, Urumqi, CHN; 2 Internal Medicine, CMH Lahore Medical College, Lahore, PAK; 3 Internal Medicine, Veterans Affairs Palo Alto Health Care System - Stanford University School of Medicine, Palo Alto, USA; 4 Internal Medicine, Endocrinology Services, Icahn School of Medicine at Mount Sinai, Queens Hospital Center, New York, USA; 5 Internal Medicine, Icahn School of Medicine at Mount Sinai, Queens General Hospital, New York, USA

**Keywords:** hhs, rhabdomyolysis, acute kidney injury

## Abstract

The hyperglycemic hyperosmolar state (HHS) is a serious acute complication of type 2 diabetes mellitus that requires prompt recognition, diagnosis, and treatment. Reversible acute kidney injury is common in hyperglycemic states. However, hyperglycemic emergencies can contribute to the development of rhabdomyolysis, which can further aggravate acute kidney injury and can cause high morbidity and mortality. HHS can be the first clinical presentation of diabetes mellitus in some patients. Here, we present a case of HHS-related rhabdomyolysis and acute kidney injury, which was the first presentation of type 2 diabetes mellitus in this patient. Our case highlights the importance of a rare association between rhabdomyolysis and HHS in diabetic patients.

## Introduction

Hyperglycemic hyperosmolar state (HHS) is a life-threatening acute metabolic complication of diabetes mellitus. Symptoms of HHS develop insidiously with polyuria, polydipsia, and weight loss, often persisting for several days before hospital admission. The rate of hospital admissions for HHS accounts for less than 1% of all primary diabetic patients [1]. The mortality rate for patients with HHS is 5%-20% and is considerably higher than diabetic ketoacidosis (DKA) [2].

Rhabdomyolysis is a syndrome resulting from necrosis of skeletal muscles leading to the release of potentially toxic intracellular contents into the circulation [3]. Rhabdomyolysis presents clinically with myalgias, red to brown urine due to myoglobinuria, and elevated muscle enzymes, including creatinine kinase [4]. Cases associated with hyperosmolarity due to hyperosmolar hyperglycemic state or DKA are described in the literature [5]. Hypophosphatemia and other electrolyte disturbances may contribute as a risk factor for rhabdomyolysis in these diabetic patients. The clinical features of rhabdomyolysis can vary from being asymptomatic to life-threatening acute renal failure requiring renal replacement therapy and a missed diagnosis can be associated with a high mortality rate.

We describe a case of rhabdomyolysis associated with HHS and complicated with acute kidney injury in a newly diagnosed type 2 diabetes mellitus patient. This case may help clinicians to improve treatment and outcomes for such patients.

## Case presentation

A 60-year-old female with no past medical history of note presented to the ED with a one-week history of lethargy, poor appetite, and frequent urination. Her family history was significant for type 2 diabetes mellitus. On admission, her blood pressure was 100/60 mmHg, her pulse rate was 120 beats/min that was regular, her respiratory rate was 30/min, and her temperature was 35.9 degrees centigrade. Chest auscultation showed bilateral vesicular breathing with a few scattered rhonchi. Neurological examination revealed no focal deficit, no signs of meningeal irritation with GCS 14 (E4M6V4). She had positive signs of dehydration, and the rest of the physical examination was not significant. The laboratory examinations revealed leukocytosis, high blood glucose, hypernatremia, elevated creatinine, hyperphosphatemia, and high anion gap metabolic acidosis, as shown in Table 1.

**Table 1:** Lab investigations at the time of admission and at the time of discharge

Labs	At the time of admission	At the time of discharge	Reference range
Serum Creatinine	5.7mg/dl	0.9mg/dl	0.61–1.24
Serum Sodium	159mEq/L	140mEq/L	136–145
Serum potassium	4.5mEq/L	3.6mEq/L	3.5–5.5
Chloride	105mg/dl	95mg/dl	96–110
Serum calcium	10.5mg/dl	8.6mg/dl	8.5–10.5
Serum phosphorus	6.3mg/dl	3.5mg/dl	2.5–4.6
Uric acid	13.8mg/dl	7.0md/dl	2.4-6.0mg/dl
BUN	160mg/dl	18mg/dl	7-20mg/dl
Calculated osmolarity	436mosm/kg		275-295
pH	7.1		7.35–7.45
pCO_2_ (mmHg)	32.6		35-50
pO_2_ (mmHg)	86		85-105
Bicarbonate (mmol/L)	13.1		22-26
Hemoglobin A1c (%)	10.7		4.5–6%
Creatinine phosphokinase ((IU)/L)	15061	230	22–232
Anion gap	40.9mEq/L		8–16
Serum glucose	1089mg/dl		7-110
LDH ((IU)/L)	600	300	91–200
Urine glucose (mg/dl)	500mg/dl		negative
Urine blood	Large		negative
Urine RBC	5-7		negative
Urine ketones (mg/dl)	20		negative
Urine protein (mg/dl)	150		negative
Urinary myoglobin	Positive		negative

**Figure 1:**
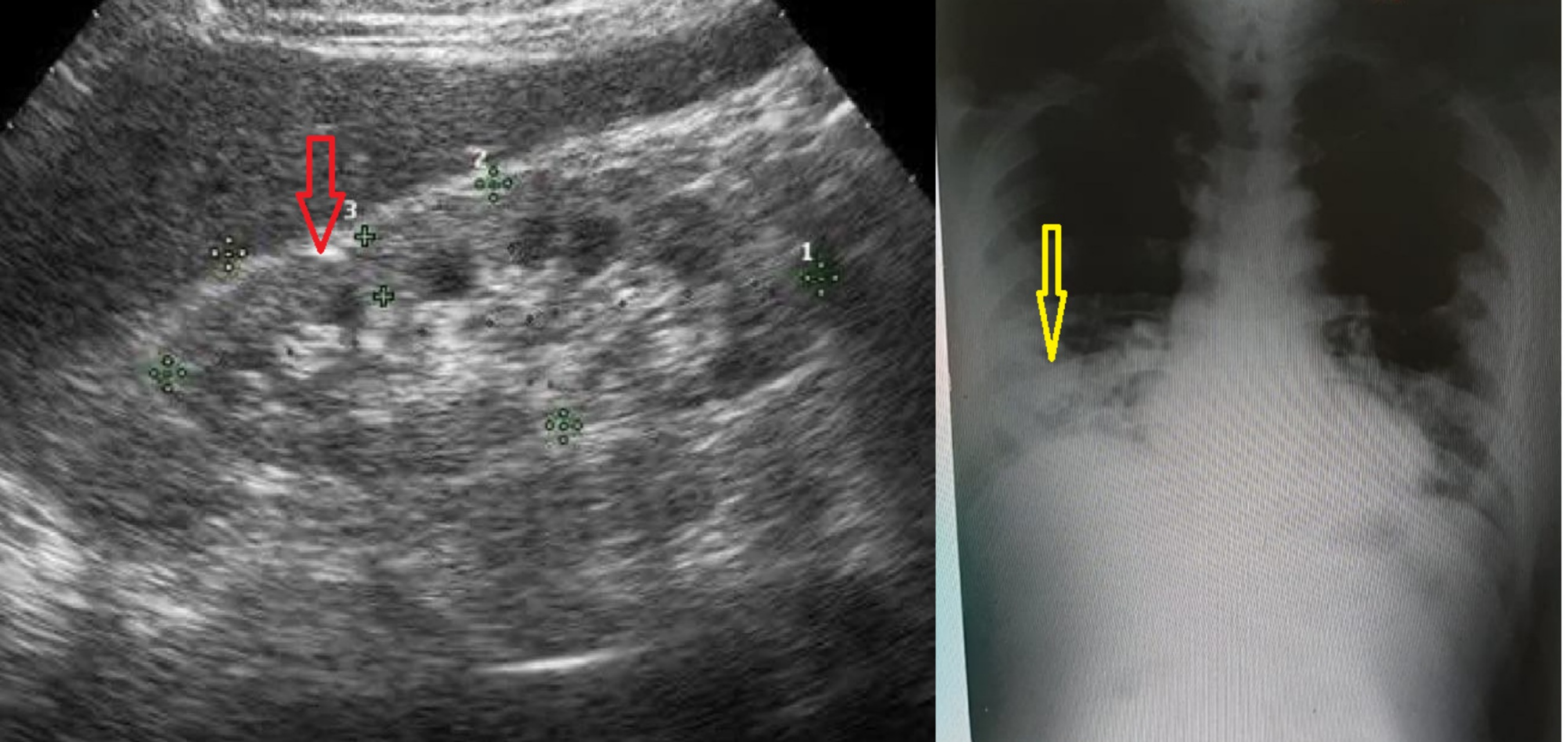
Chest x-ray (CXR) on the day of admission showed a right basal pneumonic patch (yellow arrow). Kidney ultrasound showed normal-sized kidneys with grade one echogenicity and no hydronephrosis (red arrow)

Urinalysis was positive for proteinuria, glycosuria, and significant blood, with only a few red blood cells on urine microscopy. The laboratory evaluation of lactate dehydrogenase (LDH), creatine kinase (CK), and urinary myoglobin was performed and was found to be elevated. Kidney ultrasound showed normal-sized kidneys with grade one echogenicity and no hydronephrosis as shown in Figure 1. Radiological evaluation of the chest with a chest x-ray (CXR) revealed a right basal pneumonic patch as shown in Figure 1.

**Figure 2:**
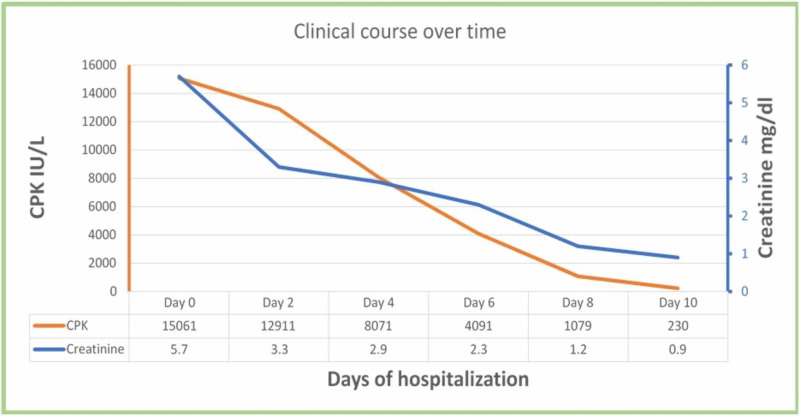
Graphical representation of creatinine and CPK levels over time from admission until recovery CPK, creatinine phosphokinase

Based on these laboratory findings, she was diagnosed with HHS, acute kidney injury, rhabdomyolysis, and pneumonia. Laboratory investigations at the time of presentation in the ER and discharge are given in Table 1. The patient was aggressively hydrated with normal saline by estimating the fluid deficit (100 ml per kg was given), and insulin therapy were initiated for the treatment of HHS. Fluid resuscitation was guided by vital signs, urine output, and improvement in sensorium, and there were no restrictions for fluid replacement. The patient was transferred to the intensive care unit (ICU) and started on empiric antibiotics for her pneumonia. Sodium bicarbonate for urine alkalization was avoided because of life-threatening hypernatremia. Her consciousness level improved in two days. After four days of treatment with continuous hydration and insulin infusion, her blood glucose, osmolarity, and creatinine phosphokinase (CPK) levels also improved significantly. After the resolution of HHS, she was transitioned to subcutaneous insulin and started on a diabetic diet. The patient was diagnosed with diabetes type 2 on this admission, with elevated hemoglobin A1c levels (10.7%). After seven days of treatment, serum creatinine and CPK were within normal limits. Subsequently, the patient was discharged from the hospital. The graphical representation of the patient's creatinine and CPK levels during the hospital stay are shown in Figure 2.

She was followed up in outpatient after two weeks, and all lab investigations were within normal limits. We have obtained informed consent from the patient for reporting this case. The authors thank the patient for agreeing to participate and for providing a detailed medical history.

## Discussion

The etiology of rhabdomyolysis can be categorized under three broad headings: traumatic, nontraumatic exertional, and nontraumatic non-exertional. The most common causes of atraumatic rhabdomyolysis are alcohol, compression, and seizure. Hyperosmolar nonketotic coma causing rhabdomyolysis is extremely rare. The association with HHS and rhabdomyolysis has been thoroughly documented as a complication of a hyperglycemic crisis. Acute kidney injury (AKI) is common and responsive to fluid infusion in hyperglycemic emergencies. In a study by Orban et al., the incidence of AKI in patients with diabetic crises (HHS and DKA) was 50% at the time of admission at the hospital [6]. The most common cause of AKI in these patients is volume depletion, which can be corrected with adequate fluid resuscitation, and renal replacement therapy is rarely needed.

The mechanism of rhabdomyolysis is a disturbance in cellular metabolism inside and outside the cells. The high osmolarity can potentially cause the breakdown of the muscle cell wall [7]. Low level of insulin or insulin resistance, as in this case, leads to decreased glucose (substrate) inside the muscle cells can cause direct attenuation of Na+/K+ ATPase activity [8]. This decreased activity will cause more Na to accumulate inside the cell and reduce the exchange of Ca with Na from intracellular space. More calcium inside the cell results in the dissolution of the muscle fibers causing myoglobin to be released into the bloodstream [9]. The myoglobin in turn blocks the tubules within the nephrons, leading to severe kidney damage. In addition to that, electrolyte abnormalities such as hypernatremia, masked hypokalemia, and hypophosphatemia (commonly seen in diabetic emergencies like hyperosmolar hyperglycemia state) also contribute to rhabdomyolysis [10].

A thorough history and physical exam are imperative to diagnosing cases of rhabdomyolysis. Highly suspected cases can be confirmed by CK and myoglobin levels. The gold standard for laboratory diagnosis is the determination of plasma CK. Although a cut-off threshold has not been established, a concentration five times the upper limit of the normal reference range (i.e., 1,000 IU/L) is commonly used [11].

Management is based on treating the underlying cause, preventing rhabdomyolysis in high-risk groups, using aggressive fluid resuscitation, administering diuretics, or alkalization, and when required, renal replacement therapy (RRT). Patients with multiple-organ-dysfunction who are hemodynamically unstable will better tolerate continuous RRT (CRRT), which rapidly controls fluid overload and electrolyte imbalances [12].

According to the American Diabetes Association, one of the diagnostic criteria for HHS is that pH remains above 7.3 in most cases [13]. Our patient had a pH of 7.1 at the initial presentation without significant ketonuria. There was positive blood on urine dipstick with few red cells on urine microscopy. The aforementioned results led to the suspicion of rhabdomyolysis. This prompted us to check CPK levels which were found to be elevated. Rhabdomyolysis induced acute renal failure has been attributed to severe volume depletion and could easily have been missed if CPK levels were not checked. This was a distinguishing feature of our case. Therefore, when presented with the appropriate clinical setting in conjunction with a hyperglycemic crisis, CPK levels should be ordered early to detect rhabdomyolysis.

## Conclusions

Type 2 diabetes mellitus can present the first time as an HHS leading to rhabdomyolysis complicated with acute kidney injury. It is crucial to make an early diagnosis due to the high mortality associated with rhabdomyolysis and HHS patients. Rhabdomyolysis should be suspected in patients presenting with hyperosmolar states due to diabetic emergencies and acute kidney injury. The early recognition of rhabdomyolysis, followed by prompt fluid resuscitation, can result in an excellent prognosis and renal recovery. Due to the rarity and clinical nature of the disease, the clinical trials are challenging to conduct. However, a better approach is required to diagnose and manage this condition.
